# The Synthesis of Selenium Nanoparticles and Their Applications in Enhancing Plant Stress Resistance: A Review

**DOI:** 10.3390/nano15040301

**Published:** 2025-02-16

**Authors:** Xin Qin, Zijun Wang, Jie Lai, You Liang, Kun Qian

**Affiliations:** 1 College of Plant Protection, Southwest University, Chongqing 400715, China; q160657@email.swu.edu.cn (X.Q.); lj0520@email.swu.edu.cn (J.L.); 2 Co-Innovation Center for Modern Production Technology of Grain Crop, Jiangsu Key Laboratory of Crop Genetics and Physiology, Yangzhou University, Yangzhou 225012, China; mz120221771@stu.yzu.edu.cn (Z.W.); liangyou@yzu.edu.cn (Y.L.)

**Keywords:** selenium nanoparticles, agriculture, plants, biotic stress, abiotic stress

## Abstract

Nanoparticle-based strategies have emerged as transformative tools for addressing critical challenges in sustainable agriculture, offering precise modulation of plant–environment interactions through enhanced biocompatibility and stimuli-responsive delivery mechanisms. Among these innovations, selenium nanoparticles (SeNPs) present unique advantages due to their dual functionality as both essential micronutrient carriers and redox homeostasis modulators. Compared to conventional selenium treatments, SeNPs offer a more efficient and environmentally friendly solution for improving plant resilience while minimizing toxicity, even at low doses. This review provides a comprehensive analysis of methods for synthesizing SeNPs, including chemical reduction, green synthesis using plant extracts, and biological techniques with microbial agents. Additionally, the review discusses the effects of SeNPs on biotic and abiotic stress responses in plants, focusing on how these nanoparticles activate stress-response pathways and enhance plant immune function. The primary objective of this study is to offer theoretical insights into the application of SeNPs for addressing critical challenges in modern agriculture, such as improving crop yield and quality under stress conditions. Moreover, the research highlights the role of SeNPs in advancing sustainable agricultural practices by reducing reliance on chemical fertilizers and pesticides. The findings underscore the transformative potential of SeNPs in crop management, contributing to a more sustainable and eco-friendly agricultural future.

## 1. Introduction

The intensification of global climate change has created unprecedented challenges for agricultural systems, with multi-stress interactions now recognized as the dominant constraint on crop productivity [1,2,3]. Beyond conventional abiotic pressures (drought, salinity, extreme temperatures) and biotic threats (pathogens, pests) [4,5,6,7,8], emerging systemic stressors, including soil degradation, micronutrient depletion, and xenobiotic accumulation [8,9,10,11,12,13,14], demand innovative solutions for ensuring food security [15,16,17,18]. In this critical context, nanotechnology has emerged as a transformative frontier, offering precision-engineered tools to reprogram plant–environment interactions through size-tunable properties and stimulus-responsive behaviors [19].

Selenium (Se), a trace element, has been extensively studied for its role in alleviating both biotic and abiotic stresses in plants [20,21,22,23]. It is essential for key physiological processes such as redox reactions and photosynthesis, improving chloroplast function and increasing photosynthetic efficiency, which ultimately leads to greater biomass production and crop yield [24,25,26,27,28]. Additionally, Se plays a crucial role in the plant’s antioxidant defense system by being a vital component of enzymes like glutathione peroxidase and selenoprotein P, which protect cellular structures from oxidative damage caused by free radicals [29,30,31,32]. Se also regulates plant hormones, such as gibberellins and auxins, which contribute to improved growth and stress adaptation [33,34,35,36]. Furthermore, Se enhances the absorption and utilization of essential nutrients like nitrogen, phosphorus, and potassium, all critical for plant development [37,38]. Through these combined actions, Se boosts plant resilience to a wide range of stresses, improving agricultural productivity and crop quality [39,40,41].

Nanotechnology has significantly improved the bioavailability of Se in plants while reducing its toxicity [42,43]. Compared to traditional Se formulations, SeNPs (SeNPs) not only facilitate easier absorption by plants but also enhance the delivery of nutritional support and antioxidant protection [44,45]. Additionally, SeNPs strengthen plant immune systems, improving resistance to pests and diseases [46,47]. More importantly, SeNPs reduce the need for chemical fertilizers and pesticides, thus enhancing crop resilience, yield, and quality, which promotes sustainable agricultural practices [48]. SeNPs can be synthesized using various methods, including physical techniques [49,50], chemical methods [51,52,53,54], and biological approaches [55,56]. Each method has distinct advantages and limitations regarding cost, scalability, particle size and shape control, and environmental impact [57]. Collectively, these techniques provide valuable resources for using SeNPs to mitigate biotic and abiotic stresses. As the application of SeNPs in agriculture expands, these nanoparticles are expected to play a pivotal role in the future of agriculture, offering essential technological support for global efforts toward sustainable agricultural practices.

**Figure 1:**
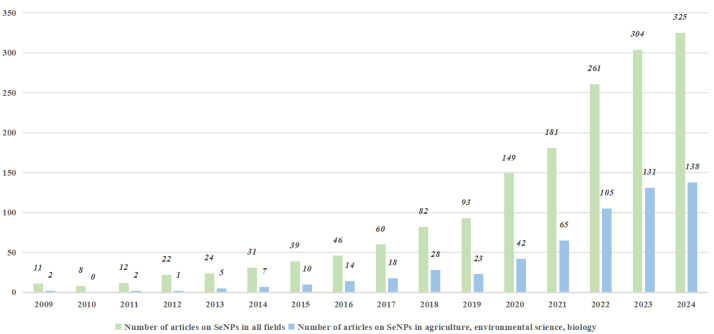
Number of articles on SeNPs in different fields (Data from web of science).

**Figure 2:**
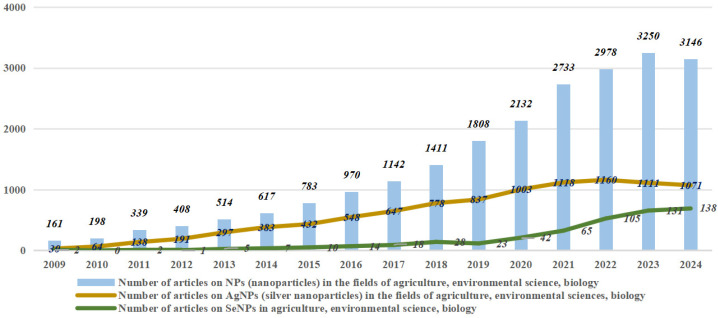
Number of articles on SeNPs, AgNPs, and NPs (Data from web of science).

In view of these excellent properties, SeNPs have been gradually attracting attention from researchers from various fields in recent years (Figure 1), but there has been relatively little research on SeNPs in agriculture-related fields, especially relative to metal nanoparticles (Figure 2). The aim of this review is to synthesize the various synthesis methods of SeNPs, to elucidate their uptake and translocation mechanisms in plants, and to explore their various applications in enhancing plant resistance to biotic and abiotic stresses. This analysis provides important insights into the safe and efficient use of SeNPs in agricultural production, which will ultimately facilitate the integration of nanotechnology into sustainable agricultural systems.

## 2. Preparation of SeNPs

**Figure 3:**
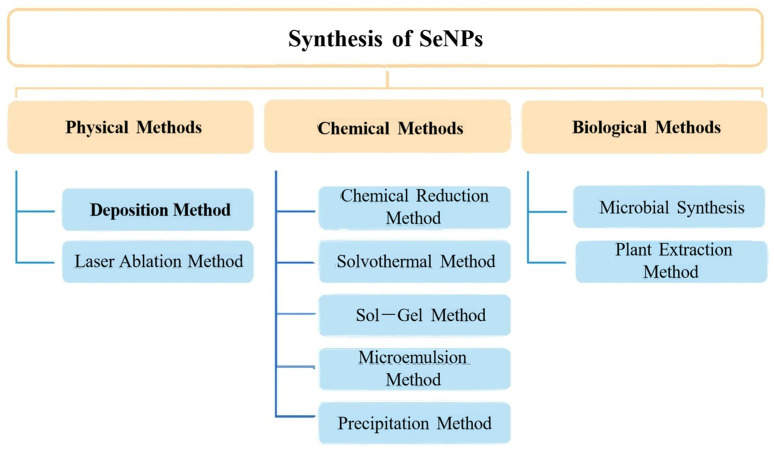
Various strategies for the fabrication of SeNPs.

**Figure 4:**
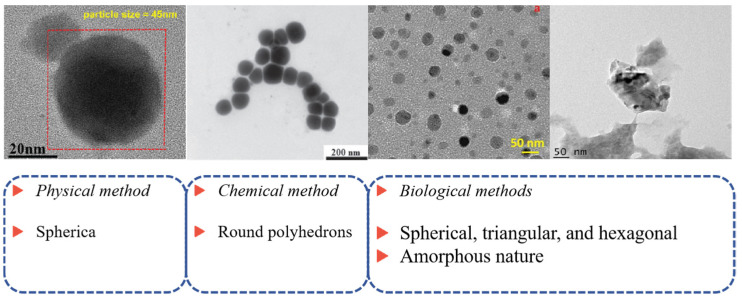
TEM images of SeNPs with different morphologies [61,62,63,64].

SeNPs are defined as Se particles with diameters between 1 and 1000 nm (typically 5–350 nm). SeNPs show better bioavailability and biological activity compared to inorganic and organic Se compounds, and generally, SeNPs with a smaller particle size have higher biological activity. Methods for synthesizing SeNP nanoparticles include physical, chemical, and biological methods, as shown in Figure 3 [58,59,60]. The choice of synthesis method depends on the specific experimental requirements and application scenarios, and SeNPs synthesized by different methods have different morphologies and sizes. Among them, SeNPs synthesized by physical methods are basically spherical, while SeNPs synthesized by chemical and green methods have various structures such as triangular, hexagonal, Round polyhedrons, and so on (Figure 4).

### 2.1. Physical Methods

#### 2.1.1. Deposition Method

Deposition is a widely employed physical method for synthesizing SeNPs, categorized into two primary techniques: chemical vapor deposition (CVD) and physical vapor deposition (PVD) [65,66]. In CVD, selenium-containing organic compounds undergo thermal decomposition at elevated temperatures, generating Se atoms or compounds that are then deposited onto a substrate, forming SeNPs [67]. In contrast, PVD involves sublimating a solid Se source into vapor, which is subsequently deposited as Se atoms or clusters on a substrate [68]. Both methods operate in high-vacuum environments to ensure purity and precision in the deposition process.

SeNPs synthesized using deposition methods have notable advantages, including ease of operation, precise control over particle size, high purity, and environmental compatibility [69]. These features make them suitable for applications in scientific research and industrial production, particularly in fields like nanotechnology, biomedicine, and materials science, where particle size and purity are critical.

#### 2.1.2. Laser Ablation Method

In the laser ablation method, high-purity Se targets, such as solid blocks or sheets, serve as the raw material [70]. A high-energy laser beam is directed at a Se target, causing localized heating and partial vaporization of the Se material. As the vapor cools and condenses, atomic clusters of Se form, which subsequently stabilize into Se nanoparticles. This method is efficient and free from chemical contamination and involves low equipment costs, making it ideal for laboratory research [71]. Liquid-assisted laser ablation (LAL), performed in solvents such as water or ethanol, has become as prevalent as atmospheric ablation. LAL confines the plasma plume within the liquid medium, enabling rapid cooling and producing smaller (5–30 nm) monodisperse SeNPs with in situ surface passivation for enhanced stability. Compared to atmospheric ablation (30–100 nm nanoparticles requiring post-synthesis stabilization), LAL also reduces energy consumption by ~40% and supports scalable production [72].

### 2.2. Chemical Methods

In general, physical methods are simpler and more convenient than chemical methods and are more capable of accurately controlling the particle size and purity of nanoparticles. However, physical methods usually require calcination in the step, which makes them unsuitable for some applications. Currently, the chemical synthesis method is the most employed one.

#### 2.2.1. Chemical Reduction Method

The chemical reduction method is a widely adopted technique for synthesizing SeNPs. In this process, Se precursors such as sodium selenite (Na_2_SeO_3_) or selenate (SeO_4_^2−^) are dissolved in a solvent, and a reducing agent—typically sodium borohydride (NaBH_4_) or ascorbic acid—is added. The reducing agent converts Se ions (Se^4+^ or Se^6+^) into elemental Se (Se^0^), which nucleates to form SeNPs [73]. This method offers simplicity and precise control over reaction parameters such as pH, temperature, and concentration, making it suitable for both small-scale research and large-scale industrial production. The ability to produce high-purity, stable SeNPs makes this approach popular in fields such as agriculture, biomedicine, and environmental management [59].

#### 2.2.2. Solvothermal Method

The solvothermal method for synthesizing SeNPs involves using a sealed reactor under high temperature and pressure [74]. In this method, the Se precursor and reducing agent are dissolved in a solvent such as water or ethanol and transferred into a high-pressure reactor, where the reaction takes place under controlled conditions. After the reaction, the reactor is cooled, and the SeNPs are isolated. This technique allows for precise control over nanoparticle size and morphology by adjusting variables like temperature, pressure, reaction time, and solvent choice [75]. However, it requires specialized equipment capable of withstanding extreme conditions, and the process can be more complex than other methods. Despite these challenges, the solvothermal method is highly effective for producing SeNPs with customizable properties.

#### 2.2.3. Sol–Gel Method

The sol–gel method synthesizes SeNPs by transforming a solution into a solid via a controlled sol–gel process [76]. In this approach, Se precursors are dissolved in a solvent, and a gelation agent (an acid or base) initiates gel formation. Hydrolysis and polycondensation reactions lead to the formation of a solid gel, which is subsequently dried and thermally treated to remove solvents, resulting in SeNPs [77]. This method is highly valued for its ability to control nanoparticle size, distribution, and morphology by manipulating parameters such as pH, temperature, and reagent concentrations [78]. It also produces highly purified SeNPs with adjustable porosity, making it suitable for applications in catalysis, drug delivery, and environmental remediation [70,79]. However, the process can be time-consuming, and careful control is needed to avoid particle aggregation.

#### 2.2.4. Microemulsion Method

The microemulsion method synthesizes SeNPs by establishing a micro-scale reaction environment within a water-in-oil system [80]. In this system, a Se precursor is dissolved in the aqueous phase, and a reducing agent is introduced to initiate the formation of SeNPs. The method provides high stability and uniformity, making it ideal for both research and specific industrial applications [81]. Nevertheless, the complexity of the process necessitates the meticulous control of reaction conditions to ensure consistency and quality. Additionally, post-synthesis purification is frequently required to remove residual surfactants and by-products, further complicating the procedure.

### 2.3. Biological Methods

Considering that chemical synthesis methods are often characterized by environmental hazards and high costs, biosynthetic methods are beginning to attract the attention of researchers. It is conceptualized as a way to reduce global, physical, and human health threats by minimizing the generation of unnecessary waste, excessive energy consumption, and cost-ineffective processes [82].

#### 2.3.1. Microbial Synthesis

**Figure 5:**
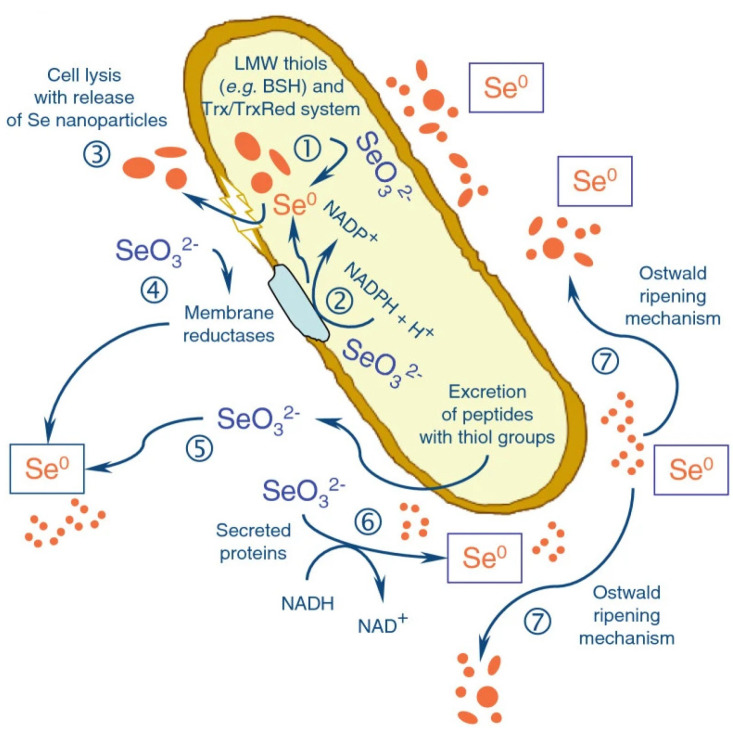
Delayed formation of zero-valent SeNPs by Bacillus mycoides SeITE01 as a consequence of selenite reduction under aerobic conditions [83].

The microbial synthesis of SeNPs is an eco-friendly method that utilizes microorganisms such as bacteria, fungi, and yeast [83,84,85]. These microorganisms act as biological reducing agents, converting Se ions (e.g., selenite or selenate) into elemental SeNPs [86]. According to the study of Silvia Lampis et al., bacterial strain SeITE01 isolated from the rhizomes of Astragalus, a selenium-hyperaccumulating legume growing in selenium-contaminated soil, reduced selenite and induced the formation of amorphous Se^0^ nanoparticles under aerobic conditions. SeNPs accumulated mainly outside the bacterial cells, and a small fraction of elemental Se was produced intracellularly [83]. This approach avoids the use of harmful chemicals and operates under mild conditions, typically at room temperature and ambient pressure [87]. However, it often requires longer reaction times, and careful optimization is needed to achieve desired nanoparticle characteristics (see Figure 5).

#### 2.3.2. Plant Extraction Method

The synthesis of SeNPs using plant extracts leverages the natural reducing agents found in plants [88]. The possibility of green synthesized SeNPs as soil antimicrobial agents was explored by Lucas et al. They mixed the extracts of three plants, *M. emarginata* (acerola cherry), *A. cepa* (onion), and *G. amygdalinum* (boldo), as reducing agents, with the precursor sodium selenite (Na2SeO), and the synthesized SeNPs exhibited bacteriostatic and bactericidal effects against Gram-positive bacteriophage [88]. These extracts, rich in bioactive compounds like polyphenols and flavonoids, reduce Se ions into elemental SeNPs [89]. Among them, algae are the most commonly used natural reducing agent because of their rapid algal reproduction rate, high biomass yield, and short culture cycle [90]. Extracts of microalgae and seaweed can be used for the green synthesis of SeCNs, and *Laminaria* polysaccharide (LP) can also be used as a stabilizer of SeCNs [91].

This method eliminates the need for harmful chemicals and operates under mild conditions, making it environmentally friendly [92]. The size, shape, and stability of SeNPs can be finely controlled by adjusting variables such as plant extract concentration, pH, temperature, and reaction time. This green synthesis method has gained attention for its potential applications in biomedicine, agriculture, and environmental remediation, though further optimization may be necessary for consistent results across different plant sources.

## 3. Plant Uptake and Transfer of SeNPs

### 3.1. Plant Uptake of SeNPs

There are two primary methods for delivering SeNPs to plants: soil application and foliar application [93,94]. Soil application as a Se fertilizer has been shown to effectively improve the Se nutritional status of crops [95]. Once in the soil, SeNPs dissolve rapidly in soil moisture, forming soluble Se compounds due to their small size and high surface area [94]. SeNPs are primarily absorbed by plant roots, the root hairs, tips, and capillaries of which facilitate their contact with the nanoparticles [96]. These particles penetrate root cell membranes and enter cells via passive diffusion or active transport mechanisms [97]. During this process, SeNPs may be encapsulated by cell membranes via endocytosis and transported throughout the plant.

Foliar application, by contrast, involves directly spraying nutrients onto leaves, offering quicker and more efficient SeNP delivery compared to soil fertilization [98]. Foliar spraying has a higher bioavailability to plants than soil fertilizer application with Se [99]. In addition, the direct foliar uptake route ensures a high degree of plant assimilation, as it does not depend on root-to-shoot translocation [100,101]. Most importantly, SeNPs at scales below 200 nm are subject to losses due to soil adsorption and chemical or microbiologically mediated transformation. The leaf surface—composed of structures like stomata, epidermal cells, and the cuticle—plays a crucial role in the absorption of SeNPs [102]. Foliarly applied nanoparticles enter the leaf through stomata and are transported to different plant parts via the exoplasmic and symbiotic pathways. Although stomata, which regulate the exchange of gases and small molecules, are not highly permeable to nanoparticles, they can still facilitate SeNP entry to some extent. Meanwhile, the cuticle and epidermal cells act as physical and chemical barriers, limiting SeNP adsorption and penetration [93]. Factors such as membrane permeability, nanoparticle surface charge, and the involvement of membrane proteins influence the entry of SeNPs into plant cells. Membrane fluidity and the role of membrane proteins are critical for nanoparticle penetration. Due to their small size, SeNPs can pass through cell membrane pores, and surface modifications can further enhance their penetration ability. The surface charge and modification of SeNPs significantly influence their absorption and transport efficiency within plants [103]. Once inside the cells, transport proteins such as sulfate transporters and ion channels aid in distributing Se throughout the plant to various tissues and organs [104].

### 3.2. Plant Transfer of SeNPs

The absorption and translocation of Se in plants initiate at the root system: selenate (SeO_4_^2−^) is actively transported into root cells via high-affinity sulfate transporters SULTR1;1/1;2 [105], whereas selenite (SeO_3_^2−^) is passively absorbed through phosphate transporters (e.g., OsPT2) and aquaporin NIP2;1 [106]. Absorbed selenate undergoes stepwise reduction in the cytoplasm: it is first catalyzed by ATP sulfurylase (APS) and APS reductase (APR) to selenite and selenide (Se^2−^) and then assimilated into selenocysteine (SeCys) via cysteine synthase (CysS) [104,107,108]. Se translocation exhibits tissue specificity: selenate is preferentially transported to shoots via the xylem transporter SULTR2;1 [109], while organic Se species (e.g., SeCys, selenomethionine SeMet) are allocated to sink tissues through the phloem-dependent amino acid transporters AAP1/LHT1 [110]. Excess Se is compartmentalized into vacuoles via SULTR4;1/4;2 [111], while chloroplasts sequester Se via SULTR3;1-4 to support photosynthetic metabolism [112]. Hyperaccumulators (e.g., *Astragalus* spp.) achieve Se hyperaccumulation through upregulated SULTRs and metabolic enzymes [113], whereas non-accumulators suffer oxidative toxicity in older leaves due to inefficient Se redistribution and growth inhibition in younger tissues from Se deficiency [114]. This process is dynamically regulated by Se-responsive transcription factors (e.g., bZIP), forming an integrated absorption–translocation–detoxification network [115].

While SeNPs hold great potential as a novel fertilizer for plant applications, current research has primarily focused on their biological effects and fundamental physical and chemical properties. However, more in-depth studies on the transformation of SeNPs within plants—particularly their conversion into various Se compounds, interactions with endogenous plant molecules, and ultimate metabolic pathways—are necessary to expand our understanding.

### 3.3. Se Toxicity Concentration

At optimal concentrations, Se not only supports plant growth and development but also enhances a plant’s ability to withstand environmental challenges [116,117,118,119]. However, while Se is highly beneficial in appropriate amounts, excessive levels can lead to toxicity, causing symptoms such as leaf chlorosis, tissue necrosis, stunted growth, and, in extreme cases, plant death [120,121].

Early on, higher concentrations of Se at 1 mg kg^−1^ were considered by researchers to be toxic to most plants. However, as the research progressed, the situation began to become more complex, and based on the available research, the exact optimal, low, and high concentrations were uncertain. These concentrations were influenced by factors such as the species of the plant, the physiological conditions of the plant, the chemical form of selenium, and the method of application, which varied greatly. The order of toxicity between the different chemical forms of selenium is elemental selenium < selenate < selenite [122]. For example, selenate concentrations between 2 µm and 10 µm have beneficial effects on growth, antioxidant capacity, and stress tolerance in hydroponically grown lettuce with a toxic threshold of 15 µm, but nitrate has a toxic threshold of 20 µm [123], and selenite and selenate have toxic thresholds of 20 µm and 80 µm for hydroponically grown cucumbers [124,125]. Se concentrations above 29 mg kg^−1^ inhibited the germination and growth of lettuce, radish, and tomato [126]. Also, the sensitivity of plants to Se varied considerably between growth stages, with plant seedlings being more sensitive to Se than mature plants. When different natural conditions are also taken into account, it becomes a more complex problem to clarify the range of beneficial concentrations and toxicity thresholds for Se. Therefore, the precise management of Se in agricultural applications is essential to avoid toxicity and maximize its benefits.

Many studies have shown that SeNPs have lower toxicity compared to other forms of selenium. For example, Li et al. illustrated that higher concentrations inhibited the growth of cabbage, which was not impeded by high doses of SeNPs (10 mg L^−1^) [43]. The possible reasons are as follows: (1) Se in SeNPs is Se^0^, and Se^0^ is less toxic than inorganic selenium [127]. (2) The slow-release property of SeNPs controls the gradual release of selenium ions (Se^2−^/Se^4^) through the surface oxidative layer [42]. (3) However, ionic selenium tends to be enriched in the root system, triggering necrosis [128].

## 4. Effects of SeNPs on Biotic and Abiotic Stresses

Biotic stress arises from interactions with living organisms, primarily pathogens such as bacteria, fungi, and viruses, as well as pests like insects and nematodes. These biological agents directly damage plant tissues, induce diseases, or compete for vital resources, ultimately compromising plant health and reducing crop yields [129]. In contrast, abiotic stress is caused by environmental factors, including extreme temperatures, drought, salinity, and heavy metal contamination [130]. These stressors disrupt critical physiological processes such as photosynthesis and metabolism, hinder water and nutrient uptake, and, in severe cases, lead to stunted growth or plant death [131]. Together, these adverse conditions severely limit crop growth, development, and productivity.

The effect of SeNPs on the stress response of animal cells has been well studied. For example, Elena et al.’s study found that SeNPs and SeNrs cause depletion of the Ca^2+^ depot of the endoplasmic reticulum and endoplasmic reticulum stress, which correlates with increased expression of genes encoding proapoptotic proteins [132]. Sergey et al., on the other hand, found that nanoparticles were able to prevent oxidative stress induced by ionizing radiation and protect animals from radiation-induced death [133]. However, studies on the effects of SeNPs on stress response in plants are still insufficiently and systematically investigated, so some of this research is collated in this paper.

### 4.1. Effects of SeNPs on Biological Stress

#### 4.1.1. Pests

Pest-induced biotic stress poses a significant challenge to agricultural production. Pests adversely affect plant growth, yield, and quality by directly feeding on crops, transmitting pathogens, and triggering plant defense responses [134,135]. With advancements in nanotechnology, SeNPs have emerged as a promising biopesticide for pest management in modern agriculture. For instance, SeNPs synthesized by Shang et al. were effective in killing Bursaphelenchus xylophilus (pine wood nematode) and exhibited lower toxicity to MC3T3-E1 cells compared to SeO_2_ [136]. In addition, Amin et al. demonstrated that SeNPs produced using metabolites secreted by a fungal strain, Penicillium, were successful in eradicating cutworms while significantly increasing the growth and carotenoid content of sunflower by 167.4% (at a concentration of 20 mg/L) [137].

While traditional pesticides help control pests, they also impose selective pressure, accelerating the development of resistance [138]. Finding novel, effective, and environmentally friendly pest control strategies is critical to addressing pesticide resistance. Zhou et al. found that foliar spraying of SeNPs effectively reduced the number of *Sitobion avenae* in wheat plants by 36.4, 26.2, and 11.5% at 5.0, 10.0, and 20.0 mg/L, respectively, compared with the control. Furthermore, the synergistic effect of SeNPs with melatonin enhances aphid resistance by promoting the synthesis of volatile organic compounds, including ethanol and acetone, and by modulating phenylpropanoid and indole metabolic pathways [139]. Arunthirumeni et al. found that SeNPs synthesized via *Trichoderma* filtrates significantly increased the mortality rate of *Spodoptera litura larvae*, with the highest antifeedant activity observed at 100 mg/L of SeNPs [140]. Other relevant examples are given in Table 1.

These findings highlight the potential of SeNPs as both larvicides and antifeedants, offering a solution to the growing problem of pesticide resistance. By exerting direct toxic effects, inducing antifeedant activity, and enhancing plant defenses, SeNPs can effectively inhibit pest reproduction and mitigate resistance development. Although challenges remain, ongoing technological advances and research suggest that SeNPs hold great promise as a green pesticide alternative in future agriculture, providing new strategies to ensure food security and promote environmental sustainability.

#### 4.1.2. Plant Pathogens

SeNPs exhibit potent antimicrobial activity [145,146], effectively inhibiting plant pathogens through multiple mechanisms. Research by Joshi et al. demonstrated that SeNPs synthesized using Trichoderma exhibited in vitro activity against *Magnaporthe oryzae*, the pathogen responsible for rice blast. At concentrations of 50 and 100 mg/L, SeNPs significantly suppressed infections caused by *Colletotrichum capsici* and *Alternaria solani* on pepper and tomato leaves, respectively [147]. Similarly, Lazcano-Ramírez et al. synthesized SeNPs using extracts from *Amaranthus glaucus* (SeNPs-AGL) and *Calendula officinalis* (SeNPs-COF), finding that at concentrations of 0.25 mg/mL or higher, these nanoparticles exhibited notable antifungal activity against Fusarium spores and *Colletotrichum gloeosporioides* [148].

One of the primary mechanisms by which SeNPs inhibit pathogens is through the induction of oxidative stress [149]. SeNPs facilitate the production of reactive oxygen species (ROS) such as superoxide anions, hydrogen peroxide, and hydroxyl radicals within pathogen cells [150]. Elevated ROS levels trigger oxidative stress, which damages critical biomolecules, including cell membranes, DNA, and proteins, ultimately leading to cellular dysfunction and death [151]. SeNPs also disrupt pathogen cell membranes through direct interactions. These nanoparticles may integrate into the membrane or bind to membrane phospholipids and proteins, altering the membrane’s physicochemical properties, increasing permeability [145,146] and leading to intracellular leakage and eventual cell death. Another key mechanism involves the inhibition of enzymes crucial for cellular respiration, such as cytochrome c oxidase or ATP synthase [150]. This inhibition reduces ATP synthesis, disrupting energy production and subsequently inhibiting pathogen growth and reproduction.

In addition to these effects, SeNPs interfere with the synthesis of cell walls [152], proteins, and nucleic acids [153]. They may induce DNA damage by causing strand breaks or binding directly to DNA, leading to structural alterations or functional disruptions. SeNPs may also destabilize RNA or interfere with the translation process, thereby impairing protein synthesis and vital cellular functions. Furthermore, SeNPs can enhance plant immune responses [154]. They stimulate the activity of defense-related enzymes, such as catalase and β-1,3-glucanase [155,156], which bolster the plant’s resistance to pathogen attacks. SeNPs also upregulate immune-responsive genes, including those encoding pathogenesis-related proteins such as PR1, PR2, and PR4, strengthening the plant’s defense mechanisms [157]. Furthermore, SeNPs also inhibit the ability of bacteria to attach to surfaces and form bacterial films and can cause bacterial death through photocatalysis [158]. Additionally, SeNPs promote the biosynthesis of phytoalexins, flavonoids, and phenolic compounds, which are crucial for defending against pathogen invasions [159,160]. Due to this combination of direct antimicrobial effects and immune-enhancing properties, SeNPs hold significant potential for controlling plant pathogens. A deeper understanding of these mechanisms will facilitate the development of more effective nanoparticle-based strategies for managing plant diseases (see Table 2).

### 4.2. Effects of Se Nanoparticles on Abiotic Stress

#### 4.2.1. Cold Stress

Cold stress severely disrupts the physiological and biochemical functions of plants, significantly impacting their growth and yield [165]. Research has demonstrated that SeNPs can notably enhance plant tolerance to cold stress through multiple mechanisms, such as increasing antioxidant capacity, protecting cell membranes, and regulating osmotic pressure. Low temperatures typically induce oxidative stress in plants, which leads to the elevated production of reactive oxygen species (ROS). These ROS attack cellular components such as lipids, proteins, and DNA, causing oxidative damage. SeNPs, with their strong antioxidant properties, effectively scavenge free radicals, thereby reducing oxidative damage at the cellular level.

Additionally, cold conditions often reduce the efficiency of photosynthesis in plants. SeNPs help to mitigate this effect by increasing chlorophyll content and promoting the activity of photosynthesis-related enzymes, which in turn improves the synthesis of photosynthetic products [166]. For example, research by Sayed et al. showed that the combined application of arbuscular mycorrhizal fungi, zinc oxide, and SeNPs significantly increased the chlorophyll content and photosynthetic rate in peppers under cold stress, ultimately leading to higher crop yields [166]. Similarly, Ramezan et al. found that under low-temperature stress, the foliar application of Se can enhance the net photosynthesis rate of cold-sensitive basil plants, protect the growth and metabolism of plants, and thus improve the growth and yield of plants under low-temperature conditions [167].

#### 4.2.2. Heat Stress

Globally climate warming has led to numerous adverse effects on plants, including cellular water loss [168], protein denaturation [169], reduced enzyme activity [170], increased oxidative damage [171], and disruptions in physiological metabolism [172]. These factors collectively impede plant growth and development and ultimately reduce yield. Enhancing plant heat tolerance to confront the challenges posed by climate change has become one of the top priorities in modern agriculture. Research suggests that SeNPs significantly mitigate the detrimental effects of heat stress on plants by reducing biomass loss [173], regulating physiological metabolism [174], boosting antioxidant defenses, and enhancing stress resistance [175].

Heat stress typically inhibits plant growth and reduces biomass; however, SeNPs alleviate these effects by stimulating growth and accelerating growth rates. Shalaby et al. found that SeNPs enhance biostimulatory effects in cucumber under saline and heat stress, increasing biomass and vegetative growth, ultimately resulting in higher yields [176]. Additionally, SeNPs regulate water metabolism in plants, improving drought tolerance and further reducing the negative impacts of high temperatures [176].

Under extreme heat conditions, SeNPs provide protective benefits for certain crops, such as sorghum, by enhancing the plant’s antioxidant defense system and protecting cells from oxidative damage. Omar et al. reported that the foliar application of SeNPs at 10 mg·L⁻^1^ under drought and heat stress conditions promotes wheat growth, improves water use efficiency, reduces membrane damage, and increases photosynthetic activity. SeNP treatment was found to improve the morphological, physiological, biochemical, and molecular traits of wheat, thus enhancing its resilience and enabling better adaptation to heat stress [174].

#### 4.2.3. Drought Stress

Studies have shown that SeNPs can significantly improve the drought resistance of plants under drought stress [177]. SeNPs can promote the growth of plant roots and increase the water absorption capacity of the roots, thereby improving the water status of plants. Ikram et al. studied the effects of different concentrations of SeNPs on wheat plants. They found that applying 30 mg/L of SeNPs at the three-leaf stage of wheat can significantly increase plant height, shoot length, shoot fresh weight, shoot dry weight, root length, root fresh weight, root dry weight, leaf area, leaf number, and leaf length [178]. SeNPs can enhance the antioxidant system in plants, including increasing the activity of superoxide dismutase (SOD), catalase (CAT), and ascorbate peroxidase (APX), thereby alleviating drought-induced oxidative damage [174,177]. SeNPs can also serve as seed-priming agents, and SeNP-mediated tomato seed priming can enhance antioxidant defense and confer drought resistance. Ishtiaq et al. tested the use of different concentrations of SeNPs as seed priming agents in a tomato field trial. The results showed that SeNP-mediated seed initiation in tomato enhances antioxidant defenses and confers drought tolerance, which could improve tomato crop growth under drought stress [179]. SeNPs can not only improve the drought tolerance of plants but also regulate the growth and development of plants [180]. Under drought conditions, SeNPs can promote photosynthesis in plant leaves and increase chlorophyll content, thereby improving the photosynthetic efficiency of plants [181]. At the same time, SeNPs can regulate hormone levels in plants; for example, they increase the content of abscisic acid (ABA). ABA is one of the main signaling molecules in plants under drought stress. SeNPs regulate the synthesis and signal transduction pathways of ABA, promote ABA accumulation, and cause stomatal closure, which helps plants retain water and reduce transpiration under drought conditions. Under drought conditions, plants accumulate osmotic regulating substances to maintain cellular water balance. SeNPs can promote the synthesis of osmotic regulating substances (such as proline, soluble sugars, and sugar alcohols) [182], helping plants maintain cellular osmotic pressure under drought stress. In addition, at the molecular level, SeNPs can upregulate stress resistance transcription factor genes (such as DREB, NAC, WRKY) [37], enhance the stress resistance of plants, and help plants better cope with drought stress.

#### 4.2.4. Salt Stress

Salt stress, particularly soil salinization, represents a significant global constraint on crop production, severely impairing plant growth, development, and yield [183]. High salt concentrations not only induce water deficits in plants but also disrupt ion homeostasis by disturbing the balance between sodium (Na^+^) and potassium (K^+^) ions [184]. SeNPs alleviate these effects by regulating the expression of Na^+^/H^+^ antiporters (e.g., NHX) and K^+^ channel proteins (e.g., AKT1), thus reducing Na^+^ accumulation, enhancing K^+^ uptake, and maintaining K^+^/Na^+^ balance [119]. Salt stress also increases the levels of reactive oxygen species (ROS) in plant cells, leading to the accumulation of superoxide anions (O_2_^−^) and hydrogen peroxide (H_2_O_2_). This oxidative stress triggers lipid peroxidation in cell membranes, damages proteins and DNA, and ultimately induces cell apoptosis. Numerous studies have demonstrated that SeNPs enhance the activity of antioxidant enzymes in crops such as wheat, bitter melon, and common beans under salt stress [119,185,186], thereby reducing ROS accumulation and protecting cells from oxidative damage. Moreover, salt stress depletes cellular water [187], necessitating the synthesis of osmotic regulators to maintain cellular water balance. SeNPs promote the accumulation of osmotic regulators such as proline, soluble sugars, and betaine, which help plants maintain osmotic pressure under saline conditions [119]. By upregulating genes involved in proline synthesis, such as proline synthase (P5CS), SeNPs further enhance osmotic regulation, mitigating the water loss caused by salt stress [188]. Additionally, SeNPs modulate the expression of salt stress-responsive genes. They activate transcription factors associated with salt tolerance, such as those from the MYB and AP2/ERF families, promoting the expression of salt-resistance genes and ultimately improving plant tolerance to salinity [189].

#### 4.2.5. Heavy Metal Stress

The excessive accumulation of heavy metal pollutants in the environment, resulting from factors such as industrial emissions, waste pollution, the improper use of pesticides and fertilizers, and natural disasters, has led to significant environmental contamination [190,191,192]. As plants grow, increased concentrations of heavy metals in soil and water induce physiological and biochemical damage, adversely affecting plant growth, nutrient uptake, and metabolic processes. This stress manifests in symptoms such as stunted growth, chlorosis, leaf curling, and wilting and, in severe cases, may result in plant death [193,194]. Studies have demonstrated that SeNPs can positively influence the physiological, biochemical, and molecular responses of plants under heavy metal stress [195,196]. Physiologically, SeNPs have been shown to increase both fresh and dry weight in plants. For instance, Zhu et al. reported that SeNPs enhance root activity, protect chlorophyll, inhibit heavy metal translocation, promote the absorption of essential nutrients, and activate the antioxidant system. These effects collectively improve the tolerance of Brassica chinensis to heavy metals and enhance its yield [197]. At the biochemical level, SeNPs bolster the plant’s antioxidant system by increasing the activity of key enzymes, such as catalase (CAT), superoxide dismutase (SOD), and glutathione peroxidase (GPx) [198]. Molecularly, SeNPs upregulate genes related to antioxidant defense (e.g., SOD, CAT) and heavy metal detoxification (e.g., MTs, PCS) [150,199], thereby enhancing the plant’s capacity to tolerate heavy metal stress. Furthermore, SeNPs form complexes with heavy metal ions, reducing their bioavailability [194]. These selenium-heavy metal complexes are less likely to be absorbed by plants or transported to vital tissues. Instead, plants can excrete the chelated heavy metal–selenium complexes, effectively lowering the free concentration of toxic metal ions and mitigating their detrimental effects. For example, research by Di et al. found that SeNPs inhibited cadmium accumulation in pak choi, reducing cadmium concentrations by 25.9–42.4% and decreasing Cd absorption rates by 33.4–37.8% following SeNP treatment in the soil. Additionally, other studies have shown that SeNPs improve the uptake of essential trace elements, such as iron (Fe), manganese (Mn), and zinc (Zn), which play critical roles in plant growth and development [200]. This enhanced nutrient absorption promotes the recovery of plants from heavy metal stress. Table 3 provides an overview of the applications of SeNPs in heavy metal sequestration.

## 5. Outlook

(1) Although SeCNs have received considerable attention as a potential agrochemical tool, their widespread application still faces several challenges.
(2) First of all, there is no clear answer to the difference between SeNPs and traditional Se products in environmental potential toxicity. Chen et al.’s study suggests that nano-sized Se may be more easily attached to the chorionic membrane of fish embryos, leading to the accumulation of drug overdoses and increasing the risk of toxicity [208,209].
(3) Second, although some studies have shown that the transport capacity of SeNPs in plants is lower than that of selenite, the specific metabolic and biological transformation processes of SeNPs still need to be further explored [43].
(4) Third, in the context of health risks associated with the human body, although some experiments have demonstrated that SeNPs exhibit lower cytotoxicity compared to organic Se and inorganic salt forms of Se—particularly in studies involving semen, in which SeNPs showed positive effects—there remain widespread concerns [210,211,212]. The potential health risks associated with SeNPs require further investigation using animal models.
(5) Finally, in order to achieve the wide commercial application of SeNPs, ensuring the stability and uniformity of product particle size is crucial, especially during the long-term transportation and storage process. Whether the nanostructure of SeNPs will change significantly is still an urgent question to be answered.

## 6. Conclusions

Nanotechnology provides an innovative and efficient way for agricultural practice to improve crop productivity and cope with environmental challenges. This review summarizes the various preparation methods of SeNPs; the absorption, transport, and conduction mechanisms of SeNPs; and the role of SeNPs in enhancing plant resistance to biotic and abiotic stresses. SeNPs have shown significant effects on plant resistance to biotic and abiotic stresses. In terms of biotic stress, SeNPs effectively improve plant resistance to pathogens and insects by enhancing the immune system, regulating disease resistance-related genes, promoting the accumulation of defense compounds, and improving the antioxidant system. In terms of abiotic stress, SeNPs alleviate the effects of cold, high temperatures, drought, salt, and heavy metal stress on plants by removing ROS, chelating heavy metals, improving osmotic balance, promoting plant growth, and improving water use efficiency. It is hoped that this article can provide a reference for agricultural producers and researchers to guide them to use SeNPs rationally and effectively in practice to improve agricultural production efficiency and sustainable development.

## Figures and Tables

**Table 1:** Antimicrobial activity of biologically synthesized SeNPs as insecticides.

SeNPs Synthesis Source	Size Range	Target Organism(s)	Effective Concentration	Reference
*Penicillium chrysogenum*	44.0–78.0 nm	*Bulinus alexandrina* (snails) *Schistosoma mansoni larvae*	5.96 mg/L 57.85 mg/L	[141]
*Clausena dentata*	46.3–78.9 nm	*Anopheles stephensi* *Aedes aegypti* *Culex quinquefasciatus* (mosquitoes)	240.71 mg/L 104.13 mg/L 99.60 mg/L	[142]
*Cupressus sempervirens*	21.0–75.0 nm	*Culex pipiens* (mosquitoes)	28.25 mg/L	[143]
*Trichoderma* sp. culture filtrate	40.0–100.0 nm	*Spodoptera litura* (tobacco cutworm larvae)	39.74 mg/L	[140]
*Trichoderma viride* fungal culture	25.4–80.6 nm	*Culex pipiens* (mosquitoes)	39.20 mg/L	[144]

**Table 2:** Antimicrobial activity of biologically synthesized SeNPs against plant pathogenic microorganisms.

SeNPs Synthesis Source	Particle Size (nm)	Target Pathogen	Effective Concentration	% of Inhibition	References
*Trichoderma atroviride* (fungus)	60.45–123.16	*Pyricularia grisea*, *Colletotrichum capsici*, *Alternaria solani*	200 mg/L	52.33	[147]
*Calendula officinalis* (flower extract)	40.00–60.00	*Colletotrichum gloeosporioides*	-	-	[161]
*Melia azedarach* (leaf extract)	74.43	*Bipolaris sarokiniana*	300 mg/L	48.31	[162]
*Amphipterygium glaucum* (leaf extract)	20.00–60.00	*Fusarium oxysporum*	300 mg/L	60.00	[163]
*Bacillus megaterium* (bacterial culture)	29.72–74.36	*Rhizoctonia solani*	1 mM	89.60	[164]
*Lactobacillus acidophilus ML14* (bacterial strain)	65.00–88.00	*Fusarium culmorum*, *Fusarium graminearum*	-	-	[46]

**Table 3:** Examples of SeNP applications for conferring heavy metal stress tolerance in different plant species.(↑ indicates growth, ↓ indicates decline).

Heavy Metal(s)	Plant (Scientific Name)	Alteration in Plant Parameters	References
As	*Brassica napus* (*Oilseed rape*)	↓ MDA ↓ H_2_O_2_ ↑ Photosynthetic rate ↑ SOD activity	[201]
As	*Pleioblastus pygmaeus* (*Dwarf bamboo*)	↑ Proline ↓ Lipid peroxidation ↓ Electrolyte leakage	[202]
Cd	*Brassica napus* (*Oilseed rape*)	↓ Respiratory burst activity ↓ ROS ↑ Glutathione ↑ Biomass	[150]
Cd	*Coriandrum sativum* (*Coriander*)	↑ Chlorophyll content ↑ Total soluble sugars ↑ Leaf RWC	[203]
Cd	*Daucus carota* (*Carrot*)	↓ MDA, ↓ H_2_O_2_ ↑ Carotenoids ↑ Soluble sugars	[204]
Cd	*Dracocephalum moldavica* (*Moldavian balm*)	↑ SOD↑ CAT ↓ MDA ↓ H_2_O_2_	[205]
Cd	*Solanum lycopersicum* (Tomato)	↑ Chlorophyll a, Chlorophyll b ↑ Ascorbic acid ↑ Soluble protein	[206]
Cd	*Triticum aestivum* (Wheat)	↑ CAT, ↑ POD ↑ RWC	[195]
Cd	*Zea mays* (Maize)	↑ Net photosynthetic rate ↑ APX activity ↑ K^+^ uptake	[102]
Cd, Pb, Hg	*Brassica chinensis* (*Pak choi*)	↓ Cd uptake, ↓ Pb uptake, ↓ Hg uptake ↑ SOD ↑ CAT, ↑ POD ↓ MDA, ↓ H_2_O_2_ ↑ Chlorophyll a ↑ Biomass	[197]
Pb, Cd	*Salvia officinalis* (Sage)	↑ RWC ↑ Chlorophyll ↓ MDA	[207]

## Abbreviations

Na_2_SeO_3_	Sodium Selenite
NHX	Na^+^/H^+^ Antiporter
NIP2;1	Silicon Influx Transporter
OsPT2	Phosphate Transporter
P5CS	Proline Synthase
PCS	Phytochelatin Synthase
PR1	Pathogenesis-Related Protein 1
PR2	Pathogenesis-Related Protein 2
PR4	Pathogenesis-Related Protein 4
PVD	Physical Vapor Deposition
ROS	Reactive Oxygen Species
SeCys	Selenocysteine
SeITE01	Selenium-Reducing Bacterial Strain
SeMet	Selenomethionine
SeNPs	Selenium Nanoparticles
SeNPs-AGL	Selenium Nanoparticles Synthesized using Amaranthus glaucus Extract
SeNPs-COF	Selenium Nanoparticles Synthesized using Calendula officinalis Extract
Se^0^	Elemental Selenium
SeO_3_^2−^	Selenite
SeO_4_^2−^	Selenate
SOD	Superoxide Dismutase
SULTR	Sulfate Transporter
WRKY	WRKY Transcription Factor Family
